# Aging and Gene Expression in the Primate Brain

**DOI:** 10.1371/journal.pbio.0030274

**Published:** 2005-08-02

**Authors:** Hunter B Fraser, Philipp Khaitovich, Joshua B Plotkin, Svante Pääbo, Michael B Eisen

**Affiliations:** **1** Department of Molecular and Cell Biology, University of California, Berkeley, California, United States of America,; **2**Max-Planck-Institute for Evolutionary Anthropology, Leipzig, Germany,; **3**Society of Fellows, Harvard University, Cambridge, Massachusetts, United States of America,; **4**Genome Sciences Department, Genomics Division, Lawrence Berkeley National Laboratory, Berkeley, California, United States of America; University of Newcastle upon TyneUnited Kingdom

## Abstract

It is well established that gene expression levels in many organisms change during the aging process, and the advent of DNA microarrays has allowed genome-wide patterns of transcriptional changes associated with aging to be studied in both model organisms and various human tissues. Understanding the effects of aging on gene expression in the human brain is of particular interest, because of its relation to both normal and pathological neurodegeneration. Here we show that human cerebral cortex, human cerebellum, and chimpanzee cortex each undergo different patterns of age-related gene expression alterations. In humans, many more genes undergo consistent expression changes in the cortex than in the cerebellum; in chimpanzees, many genes change expression with age in cortex, but the pattern of changes in expression bears almost no resemblance to that of human cortex. These results demonstrate the diversity of aging patterns present within the human brain, as well as how rapidly genome-wide patterns of aging can evolve between species; they may also have implications for the oxidative free radical theory of aging, and help to improve our understanding of human neurodegenerative diseases.

## Abstract 2

Transcriptional profiles in human and chimpanzee reveal a diversity of aging patterns present within the human brain, as well as how rapidly genome-wide patterns of aging can evolve between species.

## Introduction

Despite its ubiquity and importance, aging remains a poorly understood process. This lack of understanding is due in part to the complexity of aging, which is characterized by the gradual and progressive decline of numerous physiological processes and homeostasis, eventually leading to death [1–6]. However, recent progress in aging research has made it clear that aging processes are amenable to biochemical and genetic dissection, in both humans and model organisms [3–6].

Both environmental and genetic alterations in model organisms have been found to have profound effects on aging and lifespan. In particular, dietary restriction has been found to dramatically increase the lifespan of organisms including yeast, flies, nematodes, and mammals [4,5]; the mechanism by which this intervention reduces mortality rates is still under investigation. Additionally, inactivating myriad single genes has been found to be able to significantly increase the average lifespan of model organisms [4,5], and the identification of the pathways to which these genes belong is beginning to shed light on their possible modes of action. For example, many genes implicated in regulating lifespan in the nematode *Caenorhabditis elegans* belong to the insulin/IGF (insulin-like growth factor) signaling pathway, indicating possible connections to metabolism and a state of arrested development known as “dauer” [4,5]. Another class of genes found to be involved in the aging process is related to the production and scavenging of molecules known as reactive oxygen species (ROS), thus providing genetic evidence in support of a mechanistic theory of aging known as the free radical theory [2,3].

The free radical theory of aging was first introduced by Harman almost half a century ago [2]. This theory, as well as the related “rate of living” theory proposed earlier by Pearl [1], holds that aging is at least in part due to deleterious side effects of aerobic respiration. Specifically, mitochondrial activity leads to the production of ROS that can damage many cellular components, including DNA, lipids, and proteins [3]. These ROS, such as the hydroxyl radical (OH•) and hydrogen peroxide (H_2_O_2_), are produced in large part by the mitochondrial electron transport chain. The free radical theory has garnered widespread support in recent years; in addition to the genetic evidence mentioned above, studies from a number of model organisms showing that decreasing ROS levels leads to an increase in lifespan indicate that ROS can strongly modulate the aging process [3–5].

**Figure 1:**
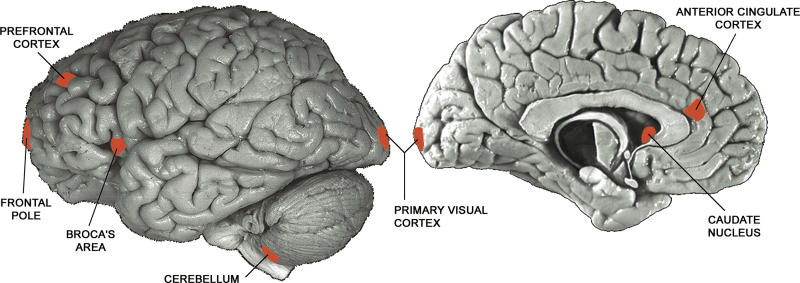
The Seven Regions of the Human Brain Analyzed in This Work The seven regions—anterior cingulate cortex, Broca's area, caudate nucleus, cerebellum, frontal pole, prefrontal cortex, and primary visual cortex—are indicated in red.

Exactly how macromolecules damaged by ROS may lead to aging has been studied in detail in recent years, and the human brain has been intensively examined in this regard because of its overall importance in human senescence. For example, up to one-third of the proteins in the brains of elderly individuals may be oxidatively damaged, and these damaged proteins have been shown to sometimes have diminished catalytic function [3,6]. One recent study of aging in the human brain demonstrated that oxidative damage to DNA can be caused by mitochondrial dysfunction, and tends to accumulate preferentially in some areas of the genome that include promoters, resulting in lower levels of transcription [7] (possibly due to loss of transcription factor or other protein binding [8–10]). In this same study, genome-wide patterns of aging-associated gene expression change in one region of the human brain cortex (the frontal pole; Figure 1) were measured using DNA microarrays, and genes that had decreased transcription with age were shown to be the ones that are most susceptible to oxidative damage [7]. Since different regions of the human brain have been shown to accumulate DNA damage at different rates [11,12], it is reasonable to suppose that these different regions may show different gene expression changes with age as a result.

Complementing studies of aging differences in various tissues within a single species, research into the evolution of aging has begun to shed light on the similarities and differences between species, although the expectation for how well conserved the effects of aging will be on a gene-by-gene basis is still unclear. One the one hand, if aging is largely caused by the deleterious effects of many alleles late in life—as is often the interpretation of two widely held models for the evolution of aging, known as “antagonistic pleiotropy” and “mutation accumulation” [4,5,13]—then rapid evolutionary change of the aging process, at least at a mechanistic level, should be impossible. This reasoning is supported by empirical observations that many aging-related factors (such as ROS-induced damage) and pathways (such as insulin/IGF signaling) appear to be highly conserved [4,5,13]. However, even if the mechanistic underpinnings of aging are indeed relatively constant, the phenotypic effects may be subject to dramatic change. This is best demonstrated by the observation that artificial selection on model organisms in the lab can lead to dramatic changes in lifespan in a very small number of generations [5,13], implying that the consequences of aging could be subject to rapid evolutionary change in the wild as well. Clearly, this issue cannot be resolved solely by laboratory evolution experiments or theoretical work.

One promising approach to answering this question of evolutionary conservation lies at the level of gene expression: Do orthologous genes tend to undergo the same patterns of expression changes with age in diverse species, or can a common factor such as ROS lead to different gene expression patterns in different organisms? Using DNA microarrays, this question can now be addressed in a systematic, genome-wide manner. One such study found that a small but significant portion of aging-related gene expression changes are shared by the very distantly related nematode and fruit fly [14]; another study comparing aging patterns in muscle cells of two more closely related species, mouse and human, also found a great deal of divergence in aging patterns [15]. Although both of these studies are informative, neither addresses the questions of how quickly age-related gene expression patterns can evolve over short periods of time, and if humans in particular show unique patterns of aging not shared by closely related primates.

The human brain is of particular interest for studying the divergence in phenotypes that have changed rapidly during evolution (such as aging). Brain-specific genes have undergone accelerated evolution in the lineage leading to human since the split with chimpanzee at the levels of both protein sequence and gene expression [16,17], pointing to the numerous functional differences that have accumulated between these two species since their divergence only 5 to 7 million years ago. Aging in the human brain is also of interest because ROS-induced damage and age are both major risk factors in many neurodegenerative diseases (such as Alzheimer's, Parkinson's, and Amyotrophic Lateral Sclerosis [18]). In this study we addressed two questions about the relationship between gene expression and aging. First, using published data, we asked whether the pattern of gene expression change with age previously observed in the frontal pole [7] is representative of other regions of the human brain. Then, using data generated for this project, we asked how similar the aging-associated changes in gene expression observed in human brain [7] are to those observed in our closest living relative, the chimpanzee.

## Results

In order to test whether different regions of the human brain show similar patterns of change with age, we utilized three independently published microarray expression datasets. These were: Lu et al. [7], mentioned above, in which the frontal pole regions of 30 individuals (aged 26–106 y) were used to identify hundreds of genes with clear up- or down-regulation associated with age; Khaitovich et al. [19], in which gene expression patterns of six brain regions (Figure 1; prefrontal cortex, primary visual cortex, anterior cingulate cortex, Broca's area, caudate nucleus, and cerebellum) were studied in three individuals (aged 45, 45, and 70 y); and Evans et al. [20], in which three brain regions (Figure 1: prefrontal cortex, anterior cingulate cortex, and cerebellum) from seven individuals (aged 18–70 y) were studied. The latter two studies were conducted to examine gene expression differences between regions of human brain; the data were not previously analyzed with respect to aging. All three studies used the same microarray platform (Affymetrix HG U95Av2), facilitating comparison between them.

### Aging Is Heterogeneous within the Human Brain

To achieve the most comprehensive picture of brain aging possible with these data, we first sought to study the patterns of aging in all six brain regions from Khaitovich et al. [19]. Because only three samples of two ages were available for each brain region in this dataset, only three general aging patterns were possible: up-regulation (the old sample is more highly expressed than either young sample), down-regulation (the old sample is more weakly expressed), or neither (the old sample is in between the young samples). Because thousands of genes would be expected to show each of these three patterns even in the absence of any genuine aging-related changes in gene expression, we were unable to use the three samples on their own to accurately identify genes changing expression with age.

However, with the available data we could ask whether the genes whose expression changes with age in frontal pole [7] showed the same direction of change in each of six other brain regions [19]. In order to do this, we reanalyzed the data of Lu et al. [7], and identified 841 genes that showed a significant (*p* < 0.01) Spearman rank correlation between age and expression level in frontal pole, irrespective of their fold change in expression; most of these were expected to be true positives, because only approximately 126 genes would be expected to pass this significance threshold by chance (corresponding to an estimated false discovery rate [21] of 126/841 = 15.0%). We classified these 841 genes as having either increasing or decreasing expression with age in frontal pole, and then as either increasing, decreasing, or constant in each of the six other brain regions. After discarding genes with no direction of change within each of the six brain regions, because these lack any information about aging changes, we tested how well the frontal pole data agree with the data from each of the six other regions. For example, comparing prefrontal cortex to frontal pole, we asked how many genes belong to each of four categories: (1) up-regulated in frontal pole and down-regulated in prefrontal cortex; (2) down-regulated in frontal pole and up-regulated in prefrontal cortex; (3) up-regulated in both regions; and (4) down-regulated in both regions. If the datasets showed similar aging patterns, we would expect an excess of genes in the latter two categories, whereas no such excess would be expected in the absence of a shared pattern. There are a number of statistical tests that can be used to quantify these patterns; we chose to use the nonparametric Spearman rank correlation coefficient (abbreviated as *r*). Values of *r* close to one indicate good agreement between aging patterns, whereas those close to zero indicate a lack of agreement. To assess the significance of these correlations, we randomly permuted the ages of the samples, and calculated the probability of observing a random correlation as strong as that found in the real data (see Materials and Methods).

**Figure 2:**
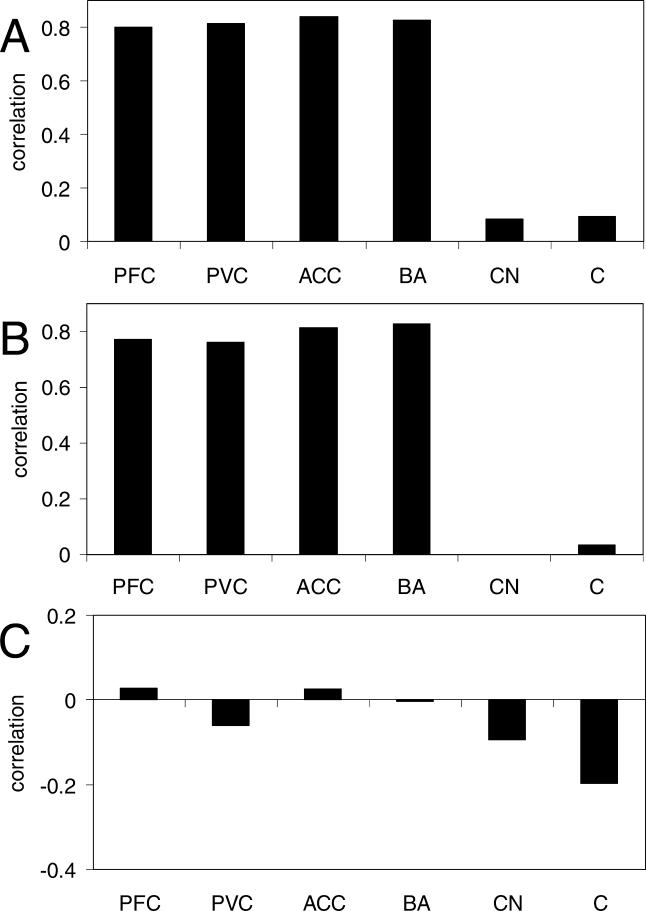
Aging in the Human Brain The abbreviations used are as follows: ACC, anterior cingulate cortex; BA, Broca's area; C, cerebellum; CN, caudate nucleus; PFC, prefrontal cortex; PVC, primary visual cortex. (A) Correlations of aging gene expression patterns between human frontal pole [7] and each of the six regions of the human brain from [19] (from left to right, number of genes used are 656, 733, 684, 710, 690, and 603). The strong correlation for all four cerebral cortex samples indicates a reproducible aging pattern across all tested regions of cortex; this pattern does not hold for caudate nucleus or cerebellum. (B) Correlations of aging gene expression patterns between human prefrontal cortex [20] and each of the six regions of the human brain from [19] (from left to right, number of genes used are 704, 832, 697, 784, 759, and 674). The strong correlations for all four cortex samples indicates a reproducible aging pattern across all tested regions of cortex but not caudate nucleus or cerebellum, confirming the result of (A). (C) Correlations of aging gene expression patterns between cerebellum [20] and each of the six regions of the brain from [19] (from left to right, number of genes used are 213, 241, 204, 241, 244, and 204). The lack of any significant correlation, even when comparing the two cerebellum aging patterns to each other, suggests that human cerebellum lacks a reproducible aging pattern.

Strikingly, all four regions of cerebral cortex for which we had expression data (prefrontal cortex, Broca's area, primary visual cortex, and anterior cingulate cortex) showed excellent agreement with the aging pattern in frontal pole (Figure 2A; *r* > 0.8 and *p* < 0.02 for each). We note that the true similarity of aging patterns in these regions is likely to be even stronger than is indicated by the correlations because, as mentioned above, approximately 15% of our genes are expected to be false positives with no true aging-related changes. In sharp contrast to cortex, the cerebellum and caudate nucleus showed far less agreement with frontal pole (Figure 2A; |*r*| < 0.1 and *p* > 0.4 for each). These results have several implications. First, the agreement between frontal pole and four regions of cortex indicates that we were able to accurately measure the direction of gene expression changes with age for most genes, even with only three samples from each region; thus the age range, number of samples, etc., are all sufficient to reflect the pattern of gene expression changes previously reported in frontal pole [7]. Second, we can have even greater confidence in the results from frontal pole [7], because they have been independently reproduced (albeit in different brain regions). Third, and most importantly, the human brain appears to have different aging patterns in cerebellum and caudate nucleus than in cortex. The fact that our four cortex samples all show strong correlations with frontal pole is akin to having a positive control, and it allows us to interpret the lack of correlation in cerebellum and caudate nucleus as evidence suggesting a difference in aging patterns, as opposed to several more trivial explanations (e.g., too few samples).

In order to further test the similarity of aging patterns within the brain, we compared a third independent dataset to the data from Lu et al. [7] and Khaitovich et al. [19]. As described above, Evans et al. [20] sampled three brain regions from each of seven individuals. We first tested whether the aging patterns in the two cortex regions from Evans et al. [20] correlated more highly with the frontal pole aging changes [7] than did the cerebellum samples, as would be expected from Figure 2A. Classifying the same 841 genes showing significant change with age in the frontal pole as either up-regulated or down-regulated with age in each brain region of this new dataset, we found the same general pattern of correlations as with the data from Khaitovich et al. [19]: Cerebellum showed a weaker correlation with frontal pole than did either cortex sample (prefrontal cortex, *r* = 0.70; anterior cingulate cortex, *r* = 0.61; cerebellum, *r* = 0.38). Although the cerebellum correlation is stronger here than in Figure 2A, it is still not significantly different from zero (*p* = 0.17), even though the two cortex samples are both significant (*p* < 0.01 each). This finding supports our conclusion that cerebellum ages differently than cortex.

For a third test of aging patterns throughout the human brain, we determined the correlation of aging patterns between a single cortex region from Evans et al. [20] with all six of the brain regions from Khaitovich et al. [19]. We used the prefrontal cortex samples from Evans et al. [20] because, as mentioned above, this brain area shows a better agreement of aging patterns with frontal pole than does anterior cingulate cortex. To facilitate comparison with cerebellum (see below), we extended this analysis to all 12,558 probe sets present on the microarray; however in order to increase the signal/noise ratio, we then excluded genes with no apparent aging changes (age vs. expression |*r*| < 0.5) in either dataset. This comparison showed the expected reproducibility of aging patterns across all four regions of the cortex: *r* > 0.76 for all four (Figure 2B; *p* < 0.03 for each except for prefrontal cortex, for which *p* = 0.067). In contrast, neither cerebellum nor caudate nucleus showed a significant correlation (Figure 2B; |*r|* < 0.04 and *p* > 0.4 for each), as expected from their lack of correlation with the frontal pole data shown in Figure 2A. In addition to providing further support for our finding of an aging pattern common to all tested regions of cortex, this result demonstrated that even when comparing aging patterns from the two smaller microarray studies used here [19,20], the age range, number of samples, etc., were sufficient to reveal a correlation when one exists.

The lack of correlation between the aging pattern in cerebral cortex with those in cerebellum and caudate nucleus might arise if the quality of data in the cerebellum and caudate nucleus samples was lower than that of the cortex samples from both Khaitovich et al. [19] and Evans et al. [20], because lower quality of data would lead to weaker correlations. To address this possibility, we first compared the expression levels of the 841 genes used in Figure 2A in the two 45-y-olds from Khaitovich et al. [19], because their equal age controls for the fact that we expect these genes not to have a very high correlation between sample of different ages (such as between the 45- and 70-y-olds). All six brain regions had highly reproducible expression levels; the lowest correlation among all six was for anterior cingulate cortex, with *r* = 0.952. The cerebellum data from Evans et al. [20] was of similarly high quality: Among five replicates of the same cerebellum samples analyzed in two different laboratories, the lowest correlation of expression levels among all genes was *r* = 0.964. Thus differing data quality could not explain the lack of correlation in cerebellum and caudate nucleus.

### Human Cerebellum Ages Less than Cortex

There are two possible explanations for the difference in the aging patterns between cerebellum/caudate nucleus and cerebral cortex. One is that cerebellum and caudate nucleus have their own aging patterns distinct from that in cortex. The other possibility is that cerebellum and caudate nucleus are different from cortex because they each have far *fewer* genes changing expression with age than cortex does, and they thus lack a reproducible pattern of aging-associated gene expression changes altogether.

To distinguish between these possibilities, one could attempt to calculate exactly how many genes change expression with age in each region; if cerebellum and/or caudate nucleus have aging-related changes in as many (but a different set of) genes than cortex, then the number of genes identified as changing in cerebellum and/or caudate nucleus should be comparable to any region of cortex. Unfortunately, as mentioned above, there is not enough statistical power to pursue this approach, given only three samples per region (or seven, as in Evans et al. [20]).

Another way to differentiate between the two possibilities listed above would be to compare two datasets of cerebellum and/or caudate nucleus aging patterns to one another. If these regions have a reproducible pattern of many genes changing expression with age (as in the cortex samples of Figure 2A and 2B), we should find a significant correlation. A comparison between the data from Evans et al. [20] and Khaitovich et al. [19] is suitable for this purpose because both datasets contain cerebellum samples and we already have a positive control that demonstrated our ability to find a correlation between aging patterns in these datasets when one exists (Figure 2B).

We thus expected to see a strong positive correlation between cerebellum aging patterns in our two datasets if and only if a large number of genes change expression with age in cerebellum. Because this analysis was carried out on all informative genes (age vs. expression |*r*| > 0.5, as in Figure 2B), instead of just the 841 with expression changes in the frontal pole, any reproducible changes in cerebellum should be found. Comparison of cerebellum aging from Evans et al. [20] with all six regions from Khaitovich et al. [19] gave an unambiguous result: Not a single region had a significant correlation (Figure 2C; |*r|* < 0.2 and *p* > 0.4 for each), including the cerebellum–cerebellum comparison. From these results, we conclude that cerebellum has a different pattern of aging than cortex because significantly fewer genes appear to change expression with age in cerebellum.

**Figure 3:**
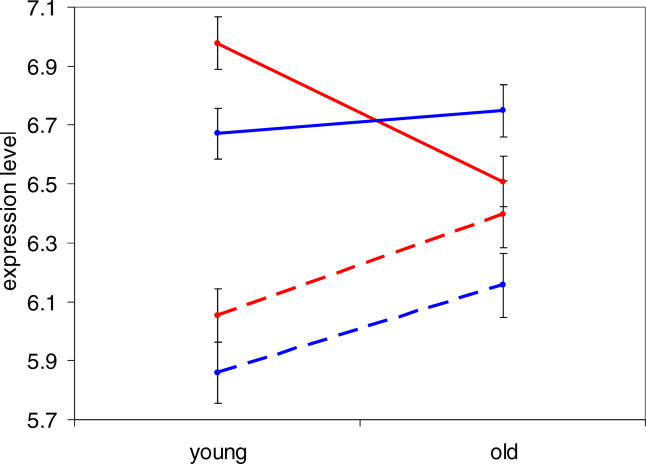
Expression Levels in Human Cortex and Cerebellum Average expression levels (base two logarithm expression intensity; error bars indicate plus or minus one standard error) in prefrontal cortex were calculated for four sets of genes in both young (two 45-y-old) and old (one 70-y-old) human samples. Red indicates cortex expression levels; blue, cerebellum expression levels; solid lines, genes down-regulated in frontal pole; and dashed lines, genes up-regulated in frontal pole (connecting lines are not meant to imply linear changes in gene expression with age). The genes up-regulated with age in cortex are somewhat up-regulated in cerebellum, whereas those down-regulated in cortex do not change at all with age in cerebellum.

In order to further characterize the differences in aging patterns between cortex and cerebellum, we calculated the average expression levels in one representative region of cortex (prefrontal cortex) for the 841 genes changing strongly with age in frontal pole, in both young (45-y-old) and old (70-y-old) samples from Khaitovich et al. [19]. As expected, when separated into two groups by their direction of change with age, clear differences were seen between the young and old samples (Figure 3). When the same genes were subjected to this analysis using their cerebellum expression levels, an interesting trend emerged: Although the genes that are up-regulated in cortex are also slightly up-regulated in cerebellum, those that are down-regulated in cortex show almost no change at all in cerebellum (Figure 3). Thus, the difference in aging patterns between these two brain regions arises mainly from genes down-regulated in the cortex. The reason for this may be related to metabolic differences between cerebral cortex and cerebellum (see Discussion).

### Chimpanzee Cortex Ages Differently than Human Cortex

In order to study the relationship between brain aging patterns in humans and chimpanzees, we required gene expression data from the chimpanzee brain. Although four studies have already produced such data, three of these [16,22,23] examined only a single brain area, and the fourth [19] had an insufficient number of samples of appropriate age for our purposes. Therefore, we generated new data by measuring gene expression levels in three regions of the chimpanzee brain: prefrontal cortex, anterior cingulate cortex, and cerebellum (see Materials and Methods). We had samples of all five regions from five individuals (aged 7 to approximately 45 y; see Materials and Methods), as well as one additional cerebellum sample and two additional prefrontal cortex samples (although excluding the three extra samples made little difference in the analysis; see Materials and Methods). Because of the very high sequence similarity between humans and chimpanzees [24], we were able to use microarrays designed for human sequences. Because we are only comparing chimpanzee samples directly with one another (comparisons with human are using only aging patterns, not actual expression levels), masking of microarray probes containing DNA sequence differences between human and chimpanzee was not necessary (and did not affect the analysis when tested).

**Figure 4:**
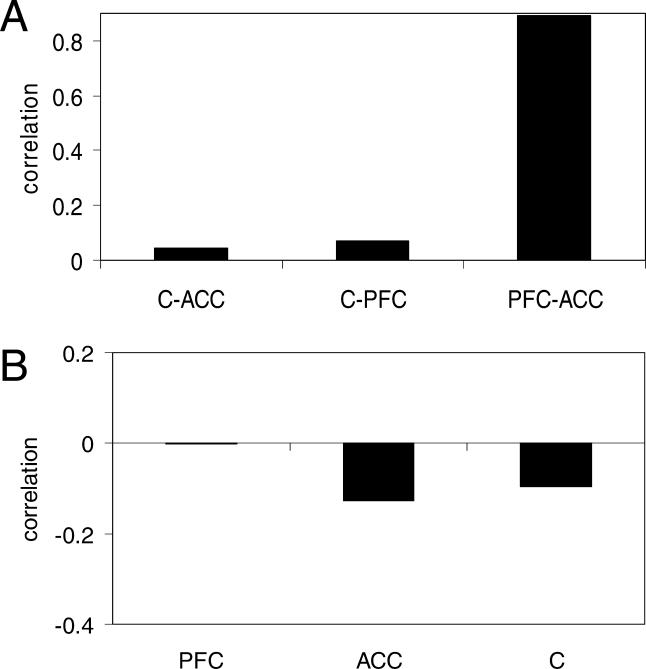
Aging in the Chimpanzee Brain The abbreviations used are as follows: ACC, anterior cingulate cortex; C, cerebellum; PFC, prefrontal cortex. (A) Correlations of aging gene expression patterns between all three possible pairs of the three regions of the chimpanzee brain used in this work (from left to right, number of genes used are 1,343, 2,235, and 1,328). The strong correlation when comparing cortex regions indicates a reproducible pattern of aging in chimpanzee cortex. (B) Correlations of aging gene expression patterns between human frontal pole [7] and each of the three regions of the chimpanzee brain used in this work (841 genes used in each comparison). The lack of any significant correlation suggests that human and chimpanzee brain aging patterns differ.

We compared the aging patterns of different regions within chimpanzee brains by applying the same methods as for comparison between aging patterns of different regions within the human brain. As in Figure 2B and 2C, we used all informative genes (age vs. expression |*r*| > 0.5) present on the microarray. Although we found no significant correlation when comparing cerebellum to either cortex region (Figure 4A; |*r*| < 0.07 and *p* > 0.3 for both comparisons), we found a very strong agreement when comparing aging patterns of prefrontal cortex to anterior cingulate cortex (Figure 4A; *r* = 0.894, *p <* 0.005). This result is precisely analogous to our findings in human, where the entire cerebral cortex shares a single pattern of gene expression changes with age that is not found in the cerebellum (see Figure 2A and 2B). Importantly, this also demonstrates that our chimpanzee samples are of sufficient number, quality, and age range to detect a correlation of aging patterns when one exists.

We then tested whether brain aging in chimpanzee is similar to that of human. Using the 841 genes that change expression with age in human frontal pole [7], we tested the agreement between the aging-related changes in frontal pole and the changes in each of our three chimpanzee brain regions. As can be seen in Figure 4B, none of the three regions showed any significant correlations with human frontal pole (|*r*| < 0.13, *p* > 0.4 for all three). Similar results were found when comparing chimpanzee aging changes in any brain region to the patterns from either of our other two human expression datasets [19,20] for either the 841 genes or all aging-informative genes on the microarray (not shown). Therefore, we conclude that chimpanzee cortex has a reproducible pattern of aging-associated gene expression changes, but this pattern is completely different from that of human cortex. Alternative explanations such as lower chimpanzee data accuracy, insufficient chimpanzee age range, and very few age-related changes in chimpanzee cortex (as in human cerebellum) can all be eliminated because they would all preclude the strong correlation between aging patterns of the two chimpanzee cortex regions shown in Figure 4A (other possible artifactual explanations for the difference are discussed in Materials and Methods).

**Figure 5:**
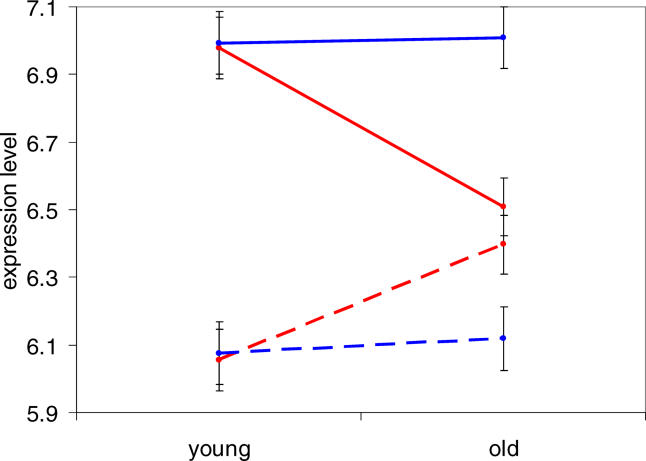
Expression Levels in Human and Chimpanzee Cortex Average expression levels (base two logarithm expression intensity; error bars indicate plus or minus one standard error) in prefrontal cortex were calculated for four sets of genes in both young (two 45-y-old human, or five 7- to 12-y-old chimpanzee) and old (one 70-y-old human, or two older than 40-y-old chimpanzee) samples. Red indicates human genes; blue, chimpanzee genes; solid lines, genes (or orthologs of genes) down-regulated in human frontal pole; and dashed lines, genes (or orthologs) up-regulated in human frontal pole (connecting lines are not meant to imply linear changes in gene expression with age). The chimpanzee expression levels resemble young, but not old, human.

Given this difference in aging patterns between humans and chimpanzees, we examined the expression levels of the chimpanzee orthologs of the 841 human genes that change expression with age in frontal pole in order to see if the expression levels of the chimpanzee orthologs of these genes resemble young humans, old humans, or neither. To test this, we first reanalyzed the expression data by masking all microarray probes with sequence differences between humans and chimpanzees [19]. We then calculated, for both human and chimpanzee prefrontal cortex, the average expression level for the set of genes that increase expression with age in frontal pole, as well as the average for the genes that decrease expression with age. The result is that in chimpanzee cortex, the orthologs of both sets of human genes (up-regulated and down-regulated) are expressed at the levels of their young human counterparts (Figure 5). In other words, chimpanzee cortex expression levels strongly resemble expression levels in young but not old humans, at least among the set of genes tested here. Humans then diverge from these average expression levels as they age, whereas chimpanzee gene expression levels change in an almost entirely different set of genes.

The high correlation of gene expression aging patterns between the two regions of chimpanzee cortex implied that the genes for which both regions show the same direction of change (that passed our cutoff of age vs. expression |*r*| > 0.5) are nearly all genuinely up- or down-regulated with age. Using this list of genes with a consistent aging pattern in the two cortex regions, there were 1,252 down-regulated and 700 up-regulated genes. Note that although the false-positive rate is likely to be low, we have no way to estimate the false-negative rate, so these numbers should not be interpreted as the total number of genes changing expression with age in chimpanzee cortex. Using this list of aging-associated genes, we tested for any significant enrichments of these genes in Gene Ontology annotation categories [19]. We did not find any enrichments for the set of genes down-regulated with age, although we found a number of significant enrichments for those up-regulated with age, including mitochondrial localization, protein degradation functions, and several metabolic processes (see Table S1). Interestingly, and consistent with our finding of no similarity between human and chimpanzee aging patterns, there was little overlap between these enriched groups and those previously reported for human frontal pole [7].

## Discussion

In this study, we have made three main observations: First, aging-related gene expression changes are similar throughout all five tested regions of the human cerebral cortex. Second, this pattern of human cortex aging is not found in cerebellum or caudate nucleus, and at least in cerebellum this appears to be due to far fewer genes changing expression with age. Third, although chimpanzee cortex has a reproducible pattern of expression changes with age, it shares no detectable similarity with the aging pattern in human cortex.

These conclusions raise a number of questions. For example, why does human cerebellum age differently than human cortex? We have shown that the majority of the difference is because genes down-regulated with age in cortex are not down-regulated in cerebellum (see Figure 3); because fewer genes change with age in cerebellum than in cortex (see Figure 2), it therefore follows that this is mostly due to fewer genes being down-regulated with age. What could cause fewer genes to have reduced transcription over time in cerebellum than in cerebral cortex? Cerebellum differs in many important respects from cortex; in particular, it has a lower metabolic rate than cortex in both human and rhesus macaque, regardless of age [25–27]. These observations of lower metabolic activity in cerebellum imply that if a consequence of aerobic respiration is ROS-induced DNA damage, then such damage should be greater in cortex than in cerebellum. Indeed, it has been shown that cerebellum has far fewer mtDNA (mitochondrial DNA) deletions than cortex, especially in old humans [11], and it accumulates less oxidative damage to both mtDNA and nuclear DNA than does cortex [12]. Therefore if the accumulation of DNA damage causes gene expression down-regulation [7–10,28], then we would expect to see fewer aging-related gene expression level reductions in cerebellum than in cortex. Our confirmation of this prediction is quite consistent with the theory that ROS-induced damage is responsible for gene expression changes [7,28], as well as the more general oxidative free radical theory of aging [2].

Similarly, one might ask why chimpanzee cortex ages differently than human cortex. If ROS-induced DNA damage is indeed a major cause of gene expression changes [7,28], then the aging differences could be due to differential ROS susceptibilities of orthologous loci in the human and chimpanzee genomes (although ROS damage is unlikely to directly explain the difference in up-regulated genes as seen in Figure 5, it may be indirectly responsible by down-regulating genes such as transcriptional repressors). It is difficult to know how plausible this is because we do not presently understand what factors lead to ROS damage susceptibility; regardless of the factors involved; however, it is quite possible that even the relatively few genetic (or epigenetic) differences between human and chimpanzee may be sufficient to cause drastic changes in ROS susceptibility, as is the case for other chromosomal properties such as DNA methylation [29] and recombination rate [30,31]. One possible explanation for ROS susceptibility is that promoters driving high levels of transcription are more vulnerable to ROS, perhaps because of their more accessible chromatin structure and/or lower tolerance for oxidative damage; however, although highly expressed genes are indeed more likely to be down-regulated with age in human frontal pole, expression levels are far from explaining all of the variation in aging-related changes in either human or chimpanzee (not shown). It is likely that ROS susceptibility will have to be measured in a number of chimpanzee gene promoters, as has been done for human [7], in order to discover if differential oxidative damage can explain the human–chimpanzee divergence in aging patterns.

Another implication of these results is related to the use of model organisms such as mouse, rat, and various primates as surrogates for human brain aging and neurodegeneration. The fact that even the chimpanzee, our closest living relative, has patterns of age-related gene expression changes almost entirely different than human implies that making specific inferences about human brain aging from model organisms may be difficult. This conclusion is supported by a study of brain aging in mice, in which in contrast to the results reported here for human, the cerebellum was found to contain *more* genes changing expression with age than cortex [32] (a difference that may be due to different relative metabolic rates of cortex and cerebellum in mouse compared to human). Model organisms are probably well suited for studying the mechanisms of aging (such as ROS-induced damage), which are likely to be conserved over great phylogenetic distances, but such conserved mechanisms may have species-specific outcomes at the level of individual genes. Thus, caution is warranted when trying to extrapolate the results of neurodegeneration research from model organisms to humans.

Many other questions raised by this work are still unresolved. First, how diverse are aging patterns of gene expression change in human tissues outside the brain? A recent study finding similar aging profiles of human kidney cortex and medulla regions implies that the intra-organ variability in aging patterns observed in the present work may not be found in all organs [33]. Second, do human and chimpanzee differ in their aging patterns in tissues other than brain, or is the brain a special case because of its recent rapid morphological evolution in the human lineage? It will be interesting to test this for tissues that have not undergone any obvious rapid evolution (such as liver or kidney), as well as for tissues that are likely to have been under strong positive selection (such as testes). Third, does chimpanzee cerebellum have fewer gene expression changes with age than cortex, as is the case in human? More chimpanzee data will be needed to address this question, although it seems likely that the answer will be affirmative because the greater metabolic rate of cortex compared to cerebellum is conserved to rhesus macaque [27]. Fourth, does the human or chimpanzee cortex aging pattern represent the ancestral state of this pattern for these two species, or are they both highly diverged from that state? Examination of brain aging patterns in an outgroup species, such as rhesus macaque, may help to resolve this question. Fifth, is the rapid divergence of aging patterns along the human and/or chimpanzee lineage the result of selection on the aging process itself, or is the divergence an indirect consequence of selection on other aspects of the brain, or could it even be explained by random drift alone? And finally, can we use our understanding of the similarities and differences in brain aging of humans and chimpanzees to gain greater insight into the causes of, and possible treatments for, human neurodegeneration? We believe this will be possible because investigation of how a phenomenon such as neurodegeneration emerged during evolution might well point us towards its underlying causes

## Materials and Methods

### Tissue samples and gene expression data

Human microarray data was obtained from Pritzker Neuropsychiatric Disorders Research Consortium (http://www.pritzkerneuropsych.org) and the National Institute of Mental Health (NIMH) Silvio O. Conte Center, and from ArrayExpress and GEO databases. Only the seven “Type 1” control individuals [34,35] were used from the Evans et al. [20] dataset.

Chimpanzee postmortem samples were obtained from Yerkes Regional Primate Center, Biomedical Primate Research Centre and Anthropologisches Institut und Museum, Universität Zürich. All individuals suffered sudden death for reasons other than their participation in this study and without any relation to the tissues used; time between death and preservation of brain tissue (postmortem interval) did not correlate with expression of genes that changes with age. Age and sex for all individuals are listed in Table S2. Total RNA was isolated from approximately 50 mg of frozen tissue using the TRIZol reagent according to manufacturer's instructions and purified with QIAGEN RNeasy kit (Qiagen, Valencia, California, United States) following the “RNA cleanup” protocol. RNAs were of high and comparable quality in all samples as gauged by the ratio of 28S to 18S ribosomal RNAs estimated using the Agilent 2100 Bionalyzer system (Agilent, Palo Alto, California, United States) and by the signal ratios between the probes for the 3′ and 5′ ends of the mRNAs of GAPDH and β-actin genes used as quality controls on Affymetrix (Affymetrix, Santa Clara, California, United States) microarrays. Labeling of 1.2 μg of total RNA, hybridization to Affymetrix HG U95v2 arrays, staining, washing, and array scanning were carried out following Affymetrix protocols. The samples were processed in random order with respect to age. All primary expression data generated for this study are publicly available at ArrayExpress database (http://www.ebi.ac.uk/arrayexpress/). Data were normalized using the Robust Multichip Average (RMA) method [36].

Among the chimpanzees used for this work, one was of indeterminate age, having been caught in the wild 40 y before its death (Table S2). However, because we used nonparametric rank statistics for all analyses, the exact age was irrelevant; all that mattered was whether it was older or younger than our 44-y-old chimpanzee. For the results shown it was assumed to be older, although assuming it to be younger than 44 y strengthened the results of the analyses (the correlation of chimpanzee prefrontal cortex with anterior cingulate cortex increased from 0.894 to 0.927).

Additionally, one pair of chimpanzees used were full siblings, and another pair were half siblings (Table S2), which could be problematic if aging patterns are family-specific. However one member of each related pair could be excluded from the analysis without greatly affecting the results (correlation of chimpanzee prefrontal cortex with anterior cingulate cortex decreased from 0.894 to 0.858), indicating that relatedness among chimpanzees does not alter our conclusions. Excluding one member of each related pair also left the same five unrelated chimpanzees for each of the three brain regions tested, controlling for any possible effects of unequal sample sizes from each brain region.

There are several possible artifactual explanations for our results not addressed in the main text. First, there is the possibility that gene expression changes correlated with age were caused by an unknown factor unrelated to the normal aging process. For example, if in the study by Khaitovich et al. [19] the 70-y-old had a disease that made his cerebellum and caudate nucleus appear “young” in their gene expression, but did not affect his cortex (because all four of his cortex regions showed the same reproducible pattern), then this could account for the results of Figure 2A. However in order for this explanation to also account for the similar results of Figure 2B, several elderly individuals from the Evans et al. [20] study would all have to be similarly afflicted in their cerebella as well. We found this to be extremely unlikely, because all ten individuals from these two studies were chosen in part for their lack of any known brain-related diseases [19,20].

Similarly, one possible explanation for the results in Figure 4A is that the cortexes (but not cerebella) of both of our old chimpanzees had a large number of gene expression differences compared to the young chimpanzees for a reason other than aging, such as a cortex-specific brain disease distinct from the normal aging process. As for the human subjects discussed above, this is extremely unlikely to be the case, because none of these chimpanzees had any apparent brain disease and both old chimpanzees (who are unrelated to each other) would have to be similarly afflicted to observe this effect.

If gene expression changes in primates tend to be dependent on chronological age (so that, for example, gene expression levels in a 45-y-old chimpanzee are most similar to those in a 45-y-old human), then the different patterns of expression changes with age seen in humans and chimpanzees could be caused by the different age ranges of the human (18–106 y) and chimpanzee (7 to approximately 45 y) samples. To control for this possibility, we truncated the data of Lu et al. [7] to contain only the 11 individuals with age ≤ 45 y. Recalculating the age-expression correlations for each gene and comparing them to the chimpanzee data, we found no more similarity in which genes change expression with age than when using any of our three full human expression datasets [7,19,20]. Therefore, we find it unlikely that brain aging of chimpanzees is any more similar to that of humans when controlling for chronological age.

Another possible explanation for the difference between human and chimpanzee aging patterns (Figure 4B) is that the difference is actually due to the different environments experienced by the humans and chimpanzees during their lifetimes, and is not due to any intrinsic differences between these species. Because there is no way to possibly control for this—for both practical and ethical reasons a human cannot be raised in precisely the same environment as a chimpanzee (or even another human)—all that we can rigorously conclude is that the humans and chimpanzees used for the analyses herein did experience different patterns of gene expression change with age. We note that this general concern extends to all studies comparing any human's phenotype with that of another organism.

One caveat concerning our interpretation of fewer genes having aging-associated changes in expression in cerebellum than in cortex is that the two human cerebellum datasets, although both consisting of grey matter of the cerebellum, were from different regions of the cerebellum (Khaitovich et al. [19] sampled the Vermis cerebelli, whereas Evans et al. [20] used the left lateral portion of the cerebellum); therefore it is technically possible that these regions of cerebellum each have their own reproducible aging pattern, which (if both shared no similarity either to each other or to cortex) would not be revealed by this analysis. We find this to be quite unlikely, given the very close proximity and functionally similar properties of these two regions, together with the finding that far more heterogeneous regions throughout the cerebral cortex share nearly identical aging patterns. And even if this improbable case were to be true, our conclusion of cerebellum grey matter as a whole lacking any reproducible aging pattern would still hold.

### Statistics

All correlation coefficients reported here were calculated by Spearman rank correlation, a nonparametric method that is robust to the presence of any outliers. The correlation coefficients from comparisons of aging profiles between two tissues or species (as in Figures 2 and 4) were calculated on coordinates assigned to genes in each of the following categories: up-regulated in both regions (1,1), up-regulated in one region and down in another ([1,−1] or [−1,1]), or down-regulated in both regions (−1,−1). The correlation coefficients can be interpreted as scores directly proportional to the fraction of genes with the same direction of expression change with age. Probability values were calculated by randomization of ages, given the specific genes used in any particular comparison: the fraction of randomizations with a correlation coefficient greater than or equal to the observed value is the *p*-value given. Therefore this is a one-sided test, appropriate for the question of whether we could have agreement between aging patterns as strong as those observed, just by random chance. The only exceptions to our using this one-sided test were when we stated we were testing whether the correlation coefficient was significantly different from zero (as opposed to greater than zero); in these cases the test was two-sided. This randomization test is somewhat conservative; for example, analyzing the leftmost bar (prefrontal cortex) of Figure 2A with Fisher's exact test yields a *p*-value of approximately 10^−99^, as opposed to approximately 0.015 from randomization. This large difference is due to the nonrandom structure of the expression data, which makes it more likely to observe strong correlations than would be expected in a set of random data. Finally, we note that our finding of significant agreement between aging patterns in datasets containing various numbers of microarrays does not imply that these numbers of microarrays will always yield sufficient power to find such a correlation if one exists.

## Supporting Information

Table S1 Gene Ontology Groups Enriched in the List of Genes Up-Regulated with Age in Chimpanzee Cerebral Cortex “All detected” is the number of genes on the microarray that had an annotation in each of the three Gene Ontology (GO) categories. “Detected/group” is the number of genes on the microarray that belong to the specific GO group listed. “All selected” is the number of genes up-regulated with age in chimpanzee cortex that have annotations in each of the three GO categories. “Selected/group” is the number of genes up-regulated with age in chimpanzee cortex that belong to the specific GO group listed. The uncorrected *p-*value cutoff for each GO group is 0.005.
(21 KB XLS).

Table S2 Sample Information (18 KB XLS).

## Abbreviations

GO: Gene Ontology
ROS: reactive oxygen species
